# Supplementary material for: A National Survey on the Clinical Practice Patterns of Korean Medicine Doctors for Attention Deficit Hyperactivity Disorder (ADHD) in Children and Adolescents
Source: Children (Basel). 2023 Aug 31;10(9):1490. doi: 10.3390/children10091490 (PMC10529033; doi:10.3390/children10091490)
Supplement: Supplementary file 1 [file children-10-01490-s001.zip › children-2530025-File S1.pdf]

Survey on the clinical practice patterns of Korean medicine doctors for Attention Deficit Hyperactivity Disorder (ADHD) in children and adolescents

1. Attention-Deficit Hyperactivity Disorder (ADHD) is a neurodevelopmental disorder that mainly affects inattention and hyperactivity-impulsivity, develops in early childhood, and interferes with various functional areas (home, school, etc.) am.

2. Symptoms of ADHD according to the DSM-5 diagnosis criteria are as follows.

[Inattention]

Six or more symptoms of inattention for children up to age 16 years, or five or more for adolescents age 17 years and older and adults; symptoms of inattention have been present for at least 6 months, and they are inappropriate for developmental level:
Often fails to give close attention to details or makes careless mistakes in schoolwork, at work, or with other activities.
Often has trouble holding attention on tasks or play activities.
Often does not seem to listen when spoken to directly.
Often does not follow through on instructions and fails to finish schoolwork, chores, or duties in the workplace (e.g., loses focus, side-tracked).
Often has trouble organizing tasks and activities.
Often avoids, dislikes, or is reluctant to do tasks that require mental effort over a long period of time (such as schoolwork or homework).
Often loses things necessary for tasks and activities (e.g. school materials, pencils, books, tools, wallets, keys, paperwork, eyeglasses, mobile telephones).
Is often easily distracted
Is often forgetful in daily activities.

[Hyperactivity and Impulsivity]

Six or more symptoms of hyperactivity-impulsivity for children up to age 16 years, or five or more for adolescents age 17 years and older and adults; symptoms of hyperactivity-impulsivity have been present for at least 6 months to an extent that is disruptive and inappropriate for the person’s developmental level:
Often fidgets with or taps hands or feet, or squirms in seat.
Often leaves seat in situations when remaining seated is expected.
Often runs about or climbs in situations where it is not appropriate (adolescents or adults may be limited to feeling restless).
Often unable to play or take part in leisure activities quietly.

Is often “on the go” acting as if “driven by a motor”.
Often talks excessively.
Often blurts out an answer before a question has been completed.
Often has trouble waiting their turn.
Often interrupts or intrudes on others (e.g., butts into conversations or games)

In addition, the following conditions must be met:
Several inattentive or hyperactive-impulsive symptoms were present before age 12 years.
Several symptoms are present in two or more settings, (such as at home, school or work; with friends or relatives; in other activities).
There is clear evidence that the symptoms interfere with, or reduce the quality of, social, school, or work functioning.
The symptoms are not better explained by another mental disorder (such as a mood disorder, anxiety disorder, dissociative disorder, or a personality disorder). The symptoms do not happen only during the course of schizophrenia or another psychotic disorder.

When answering the questionnaire concerning ADHD in children and adolescents, please fill out only the cases applicable to ADHD, excluding cases where other comorbidities (intellectual disability, autism spectrum disorder, etc.) are the main diagnoses.

Ⅰ. Current status of treatment for ADHD in children

1. On the basis of the last 1 year, what is the monthly average number of first-time patients with ADHD in children and adolescents?

① ≤ 5

② 6–10

③ 11–15

④ 16–20

⑤ ≥ 21

2. On the basis of the last 1 year, what is the average treatment period for ADHD patients in children and adolescents who visited the hospital?

① < 1 month

② ≥ 1 month to < 3 months

③ ≥ 3 month to < 6 months

④ ≥ 6 month to < 1 year

⑤ ≥ 1 year to < 3 years

⑥ ≥ 3 years

3. On the basis of the last 1 year, what is the average cost of treatment (copay) for children and adolescents with ADHD?

(If decoction is included, it is calculated by dividing the total cost by the number of days of treatment days.)"

① < KRW 5,000

② 5,000 or more and less than 10,000

③ 10,000 or more and less than 20,000

④ 20,000 or more and less than 50,000

⑤ 50,000 or more and less than 100,000

⑥ ≥ 100,000

4. On the basis of the last 1 year, what is the main age group of children and adolescents with ADHD receiving treatment? (Select up to 3 that apply)"

① Preschool children (1 or more and less than 7 years)

② Children in early elementary school (7–9 years)

③ Children in the upper grades of elementary school (10–12 years)

④ Middle school students (13–15 years)

⑤ High-school students and above (≥ 16 years)

5. On the basis of the last 1 year, among children and adolescents with ADHD who are being treated, what is the proportion of concurrent Western treatment for ADHD?

① 0%

② 1 or more and less than 25%

③ 25 or more and less than 50%

④ 50 or more and less than 75%

⑤ 75 or more and less than 100%

5-1. What type of treatment is mainly performed when a child and adolescents with undergoing treatment receives Western treatment at the same time? (Select up to 3 that apply)

① Medication (e.g. methylphenidate, atomoxetine, etc.)

② Educational interventions (behavior therapy in schools)

③ Cognitive Behavioral Therapy

④ Social skills training

⑤ Psychotherapy

⑥ Sensory integration therapy

⑦ EEG biofeedback (e.g. neurofeedback)

⑧ Parent and family counseling

⑨ Others (specify:)

6. During the past 1 year, during the treatment of children and adolescents with ADHD, how many cases were referred to western medical institutions? (Including cases of direct request, excluding cases of simply recommending treatment)

① 0

② 1–5

③ 6–10

④ 11–15

⑤ 16–20

⑥ ≥ 21

6-1. What was the main purpose of referral to a western medical institution during the treatment of patients with ADHD in children and adolescents?

① For diagnosis and examination of ADHD

② To prescribe Western medicine

③ For psychotherapy

④ Others (specify:)

Ⅱ. Diagnosis

1. Do you use pattern identification when treating children and adolescents with ADHD?

① It is treated by prescriptions commonly used for the disease without using pattern identification.

② Use pattern identification to treat.

1-1. When treating children and adolescents with ADHD patients according to pattern identification, what is the main diagnostic method used for children and adolescents with ADHD?

① pattern identification of Qi, Blood, Fluid, Humor, and Organ system diagnosis (氣血津液臟腑辨證) based on KM textbooks

② Six-Meridian pattern identification (六經辨證)

③ pattern identification of Sasang constitutional (四象醫學) diagnosis

④ Other (specify :)

1-2. What are the names of pattern identification used for children and adolescents with ADHD? (Select up to 3 that apply)

① kidney deficiency and liver hyperactivity (腎虛肝亢)

② dual deficiency of the heart-spleen (心脾兩虛)

③ phlegm-fire harassing the heart (痰火擾心)

④ spleen deficiency and liver effulgence (脾虛肝旺)

⑤ dual deficiency of the heart-kidney (心腎兩虛)

⑥ internal obstruction of static blood (瘀血內阻)

⑦ effulgent heart-liver fire (心肝火旺)

⑧ essence-blood deficiency (精血虧虛)

⑨ heart yin deficiency (心陰虧虛)

⑩ depressed liver qi transforming into fire (肝鬱化火)

⑪ Other (specify :   )

2. What diagnostic tool is being used for children and adolescents with ADHD? (Select up to 3 that apply)

① DSM-5

② DSM-Ⅳ

③ It is judged through clinical symptoms without using assessment tools.

④ Evaluation through referral to other medical institutions or medical records of other medical institutions

⑤ Other (specify :   )

Ⅲ. Treatment

1. What is the main treatment for children and adolescents with ADHD? (Select up to 3 that apply)

① Herbal medicine

② Acupuncture

③ Electroacupuncture

④ Moxibustion

⑤ Cupping therapy

⑥ Pharmacopuncture

⑦ Manipulation / Exercise therapy

⑧ Others (specify :   )

1-1. What types of herbal medicine formulations are mainly used for children and adolescents with ADHD? (Select up to 3 that apply)

① Compound herbal decoction

② Distillation of the compound herbal decoctions

③ Powder preparation

④ Pill preparation

⑤ Mixture of soluble granules covered by insurance

⑥ Soft extract covered by insurance

⑦ Mixture of soluble granules not covered by insurance

⑧ Soft extract not covered by insurance

⑨ Others (specify :)

1-2. What is the name of the herbal medicine prescription mainly used for children and adolescents with ADHD? (Select up to 5 that apply)

① Jwagui-eum

② Gammaekdaejo-tang

③ Ondam-tang (including Gamiondam-tang and Hwangryeonondam-tang)

④ Gwibi-tang (including Gamigwibi-tang)

⑤ Sayeok-san

⑥ Yukmijihwang-tang

⑦ Sihogayonggolmoryeo-tang

⑧ Gyejigayonggolmoryeo-tang

⑨ Eokgan-san (including Eokgan-san-gabanhajinpi)

⑩ Gamisoyo-san

⑪ Bosimgeonbi-tang

⑫ Bojungikgi-tang

⑬ Hwangryeonhaedok-tang

⑭ Jodeung-san

⑮ Others (specify :)

1-3. When treating children and adolescents with ADHD with herbal medicines, if herbal medicines are frequently used, what are they? (Select up to 3 that apply)

① Polygalae Radix (遠志)

② Rehmanniae Radix Preparat (熟地黃)

③ Poria (茯苓)

④ Acori Graminei Rhizoma (石菖蒲)

⑤ Zizyphi Spinosae Semen (酸棗仁)

⑥ Chinemydis Plastrum (龜板)

⑦ Trionycis Carapax (鱉甲)

⑧ Glycyrrhizae Radix (甘草)

⑨ Others (specify :)

1-4. What is the recommended average duration of herbal medicine treatment for children and adolescents with ADHD?

① < 1 month

② 1 month or more and less than 3 months

③ 3 months or more and less than 6 months

④ 6 months or more and less than 1 year

⑤ 1 year or more and less than 3 year

⑥ ≥ 3 years

1-5. What is the most commonly used acupuncture method for children and adolescents with ADHD? (Select up to 3 that apply)

① Meridian points acupuncture (經穴鍼術)

② Sa-am acupuncture therapy (舍巖鍼法)

③ Five element acupuncture (五行鍼法)

④ Constitutional acupuncture (體質鍼法)

⑤ Auricular acupuncture

⑥ Intradermal acupuncture (皮內鍼)

⑦ dermal acupuncture (皮膚鍼)

⑧ Others (specify :)

1-6. Which acupuncture points are mainly used for acupoints in children and adolescents with ADHD? (Select up to 5 that apply)

① PC6 (內關)

② LI4 (合谷)

③ LR3 (太衝)

④ KI3 (太谿)

⑤ GV20 (百會)

⑥ EX-HN3 (印堂)

⑦ EX-HN5 (太陽)

⑧ GB20 (風池)

⑨ GV14 (大椎)

⑩ HT7 (神門)

⑪ LI11 (曲池)

⑫ ST36 (足三里)

⑬ SP6 (三陰交)

⑭ GB34 (陽陵泉)

⑮ EX-HN1 (四神聰)

⑯ GB13 (本神)

⑰ GV26 (水溝)

⑱ Others (specify :)

1-7. What is the recommended average duration of acupuncture treatment for children and adolescents with ADHD?

① < 1 month

② 1 month or more and less than 3 months

③ 3 months or more and less than 6 months

④ 6 months or more and less than 1 year

⑤ 1 year or more and less than 3 year

⑥ ≥ 3 years

1-8. What types of Pharmacopunctures are mainly used for children and adolescents with ADHD? (Select up to 3 that apply)

① Cervi Pantotrichum Cornu (鹿茸)

② Hominis Placenta (紫河車)

③ Ginseng (山蔘)

④ Others (specify :)

1-9. What is the main method used for manual/exercise therapy for children and adolescents with ADHD?

① Chuna manual therapy

② pediatric Tuina

③ acupressure

④ Others (specify :)

Ⅳ. Perception of Korean medicine treatments

1. Based on your treatment experience, what is the overall effect of Korean medicine treatment on children and adolescents with ADHD?

① Not at all effective

② Mostly not effective

③ Moderate

④ Mostly effective

⑤ Very effective

2. Based on your treatment experience, which is the most effective Korean medicine treatment for children and adolescents with ADHD? (Select up to 3 that apply)

① Herbal medicine

② Acupuncture

③ Electroacupuncture

④ Moxibustion

⑤ Cupping therapy

⑥ Pharmacopuncture

⑦ Manipulation / Exercise therapy

⑧ Others (specify :)

3. What do you think are the advantages of Korean medicine treatment for children and adolescents with ADHD? (Select up to 3 that apply)

① Effective

② Fewer side effects

③ Economical

④ More helpful than other treatment methods

⑤ In addition to ADHD symptoms, it can improve a patient's overall health.

⑥ Other (Please specify:)

4. What do you think needs to be supplemented in Korean medicine treatment for children and adolescents with ADHD? (Select up to 3 that apply)

① Diagnostic accuracy

② Treatment cost

③ Therapeutic effect

④ Treatment period

⑤ Convenience of treatment (herbal medicine and acupuncture)

⑥ Treatment information (promotion and awareness)

⑦ Other (Please specify:)

5. What items do you think need additional education for Korean medicine doctors regarding the treatment of ADHD in children and adolescents? (Select up to 3 that apply)

① Diagnosis of ADHD

②Utilization of evaluation tools

③ Details of herbal medicine treatment

④ Details of non-herbal medicine treatment (acupuncture, manual therapy, etc.)

⑤ Utilization of psychotherapy

⑥ Other (Please specify:)

Ⅴ. Safety and effectiveness

1. How often is the efficacy evaluation performed in the treatment of ADHD in children and adolescents?

① 1 month

② 3 month

③ 6 month

④ 1 year

⑤ Other (Please specify:)

2. What are the efficacy evaluation indicators used in the treatment of ADHD in children and adolescents? (Select all that apply)

① Judgment based on clinical symptoms without specific indicators

② Numerical Rating Scale (NRS)

③ Academic Performance Rating Scales (APRS)

④ ADHD Rating Scale-Ⅳ (ADHD-RS-Ⅳ)

⑤ Conners Parents Rating Scale-Revised (CPRS-R)

⑥ Brown ADD Rating Scale (Brown ADD-RS)

⑦ Child Behavior Checklist (CBCL)

⑧ Home Situation Questionnaire-Revise (HSQ-R)

⑨ School Situation Questionnaire-Revised (SSQ-R)

⑩ Attention Diagnosis System (ADS)

⑪ Comprehensive Attention Test (CAT)

⑫ Wechsler Intelligence Scale

⑬ Other (Please specify:)

3. How often is the safety evaluation performed in the treatment of ADHD in children and adolescents?

① 1 month

② 3 month

③ 6 month

④ 1 year

⑤ Other (Please specify:)

4. What are the safety evaluation indicators used in the treatment of children and adolescents with ADHD? (select all that apply)

① Evaluation of Adverse Reactions

② Change in vital signs

③ Changes in the child's general condition

④ Blood test

⑤ Urine test

⑥ Other (Please specify:)

Ⅵ. Demographic information

1. Sex

① Male

② Female

2. Age

① ≤ 29

② 30–39

③ 40–49

④ 50–59

⑤ ≥ 60

3. Years of clinical experience

① < 5 years

② 5 years or more and less than 10 years

③ 10 years or more and less than 15 years

④ 15 years or more and less than 20 years

⑤ 20 years or more and less than 30 years

⑥ ≥ 30 years

4. Place of work

① Seoul Metropolitan City

② Busan Metropolitan city

③ Daegu Metropolitan city

④ Gwangju Metropolitan city

⑤ Incheon Metropolitan city

⑥ Daejeon Metropolitan city

⑦ Ulsan Metropolitan city

⑧ Gyeonggi-do

⑨ Gangwon-do

⑩ Chungcheongbuk-do

⑪ Chungcheongnam-do

⑫ Jeollabuk-do

⑬ Jeollanam-do

⑭ Gyeongsangbuk-do

⑮ Gyeongsangnam-do

⑯ Jeju-do

⑰ Sejong Metropolitan Autonomous City

5. Specialist training

① No

② Yes

5-1. Specialty

① Internal Korean Medicine

② Korean Medicine Obstetrics and Gynecology

③ Korean Medicine Pediatrics

④ Korean Medicine Neuropsychiatry

⑤ Korean Medicine Ophthalmology, Otolaryngology, and Dermatology

⑥ Korean Medicine Rehabilitation

⑦ Korean acupuncture and moxibustion medicine

⑧ Sasang Constitutional Medicine

6. Level of healthcare facility of affiliated institution

① Korean medicine clinics (primary healthcare institutions)

② Korean Medicine Hospital (not a university hospital)

③ university teaching Korean medicine hospitals

④ convalescent hospital

⑤ Public health center/health center branch/army

⑥ National Medical Center

⑦ Other (Please specify:)
